# Examining the relationship between personality traits, work experience, burnout, and intention to stay among home care aides in Taiwan

**DOI:** 10.1186/s12877-023-04136-1

**Published:** 2023-07-13

**Authors:** Yung-Ning Hung, Tzu-Ying Chiu

**Affiliations:** 1 Yuanshan Branch, Taipei Veterans General Hospital, Yilan County, Taiwan; 2 Department of Health and Welfare, College of City Management, University of Taipei, Taipei City, Taiwan

**Keywords:** LTC 2.0 policy, Home care aides, Intention to stay, Personality

## Abstract

**Background:**

Home care aides play an important role in providing long-term care services, but there is currently an insufficient supply of workers to meet the growing demand in Taiwan due to the increasing number of older adults and people with disabilities requiring care. There are numerous factors that influence the retention of home care aides. Previous research has indicated that taking employees’ individual personalities into account can enhance task delegation and organizational efficiency within an organization. Severe occupational burnout is likely to diminish vitality and disrupt sleep, which, in turn, can result in higher employee turnover rates. To date, no research has explored the correlation between personality traits, burnout, work experience, and the retention of home care aides. Given these gaps in knowledge, the present study aims to investigate how personality traits and occupational burnout are associated with the intention to stay among home care aides in Taiwan.

**Methods:**

The study was a cross-sectional survey that utilized purposive sampling to interview 285 home care aides in Hualien County from December 2020 to January 2021. A self-report questionnaire, administered with informed consent, was used to collect data on demographics, work experience, occupational burnout, personality, and intention to stay. The researchers utilized hierarchical regression analysis to analyze the data. All measurements exhibited high reliability and consistency, with Cronbach’s α values ranging from 0.8 to 0.9.

**Results:**

The subscales for personal burnout, work-related burnout, and client-related burnout, along with all personality scales, were highly correlated with intention to stay, except for the openness trait (p < .05). Moreover, married, full-time employment, satisfied with the promotion system and current job as home care aides on the whole, lower levels of work-related burnout, and agreeable personality type were found to be significant predictors for intention to stay (p < .05) , and the adjusted R^2^ of the model was 29.4%.

**Conclusions:**

This study has concluded that using personality traits as a criterion to select home care aides with a high level of agreeableness prior to recruitment, enhancing the professionalism and comprehensive of promotion system, and reducing work burnout are measures that may help home care service providers increase the intention to stay among home care aides.

## Background

Taiwan is among the nations with the most rapidly aging populations in the world. The population of older adults grew from 10.89 to 16.86% between 2011 and 2021 and is projected to reach 41.6% by 2070 according to the estimate released by the National Development Council [1]. This trend, along with the ongoing decline in Taiwan’s birth rates, has resulted in inadequacy of community and home care aides. Population aging indicates a continued decrease in the labor force and an increase in the dependency ratio. The rise in the population of older adults and people with disabilities p implies a relative growth in the number of individuals with long-term care (LTC) needs [2]. To address this problem, Taiwan implemented the Ten-year Long-term Care Plan in 2008, and officially launched the Ten-year Long-term Care Plan 2.0 (LTC 2.0), an updated LTC policy, in 2017 [2].

Other than the current services, the workforce providing services is a crucial part in the LTC services today. Individuals must follow one of the three pathways to work as a home care aide in Taiwan: (1) obtain a certificate of completion for the “Professional Training for Home Care Aides” program (organized by the Ministry of Health and Welfare) for at least 90 h; (2) obtain the “Class C Technician Certificate for Home Care Aides” from the Ministry of Labor; or (3) graduate from high school or vocational school with a degree in nursing, care, or a related field. Once they qualify as home care aides, they can provide services in institutions, communities, and homes, it is mandatory for home care aides to complete 120 points of continuing education courses every six years [3].

According to the 2019 statistics on senior and long-term care published by the Taiwan Ministry of Health and Welfare (MOHW), a total of 105,470 people were using home care services, accounting for approximately 60% of the total LTC service users. This demonstrates that home care services are the most common type of LTC service used at present [2]. Home care aides, in particular, play an indispensable role, and the care services they provide also directly affect the service quality of the entire LTC system. Since the implementation of LTC2.0, the number of home care aides in Taiwan increased from 10,478 to 2017 to 46,605 in 2021 [4]. In addition, statistics from the Taiwanese Ministry of Labor have indicated that the employment retention rate for those who obtained a Class C Technician Certificate for Home Care Aides increased from 67.7% to 2017 to 75.1% in 2021, three months after obtaining the certificate [5]. However, according to the MOHW statistics, a total of 110,000 people were trained and became licensed as home care aides between 2003 and 2016, among whom only 35,286 people are actually engaged in LTC services as of the end of 2015, whereas 41.2% of the trainees were never involved in LTC services [6]. In regards to the length of employment, the results of previous survey suggest that the number of home care aides leaving their job peaks “within three months” of employment, with an average turnover rate of 45.45% [7].Thus, although multiple training programs for home care aides are organized annually in Taiwan, the home care service sector has long experienced a workforce shortage.

There are a multitude of factors influencing the retention of home care aides. According to previous research, factors including low wages and few benefits, high levels of manual labor, discrimination against and lack of respect for this profession among the general public, and complex conditions in the working environment among others all affect their turnover [2, 6, 8]. Previous research has suggested that considering employees’ individual personalities can improve the delegation and organization of tasks within organizations [9]. In addition, the nature of the profession requires home care aides to spend long hours with people, while the clients they serve are mostly individuals with disabilities in need of assistance. Accordingly, whether home care aides have a personality that allows them to respond flexibly may also affect their intention to stay on the job. Nevertheless, existing literature on the correlation between personality traits and retention has largely focused on industries such as bancassurance, education, and tourism [10–12]. No research to date has explored how the personality traits of home care aide’s workers are correlated with their retention. Furthermore, prior studies have demonstrated that higher occupational burnout would affect the intention to stay [13–17].

Home care aides, being in a labor-intensive occupation, need to fulfill not only their job responsibilities, including providing personal care such as assisting with bathing, changing clothes, oral hygiene, eating, medication, back patting, limb and joint exercises, getting in and out of bed, as well as accompanying clients for walks, exercise, assisting with daily living, and other services. In addition, they are responsible for providing daily living care and household services such as washing and cleaning laundry, cleaning the living space and environment, performing household and administrative tasks, catering services, accompanying or purchasing necessary daily items, accompanying for medical appointments or contacting medical institutions, and other related home services [18, 19]. Moreover, they need to fulfill their job responsibilities and the requirements of their company such as achieving service goals, improving service quality as well as the needs of their clients [19], which often induces feelings of exhaustion and powerlessness among them [12]. Severe occupational burnout is likely to reduce vitality and affect sleep, which would in turn lead to an increase in employee turnover rates [20]. To date, no research has explored the correlation between personality traits, burnout, work experience, and the retention of home care aides. Given these gaps in knowledge, the present study aims to investigate how personality traits and occupational burnout are associated with the intention to stay among home care aides in Taiwan.

## Methodology

### Research Subjects

In this study, a cross-sectional survey was conducted using a self-administered questionnaire. To ensure that all home care service providers have stable caseload volumes and adequate experience, the following inclusion criteria for research subjects were adopted: (1) For full-time home care aides, they should be enrolled in the labor insurance scheme provided by the home care service institution they work at or satisfy the monthly working hour requirements for full-time employment stipulated by the institution. For part-time home care aides, they should work more than 10 h in a month on average (excluding the time commuting between homes of different clients); (2) they should fulfill the requirements for home care aides based on regulation of LTC services [21]; (3) they have been serving at the current unit of employment for more than three months; (4) they are able to communicate in Chinese; and (5) they agree to participate in this study. Exclusion criterion: (1) Institutions that are less than three months old should be excluded. With reference to prior studies and existing literature, the margin of error was set at 5%, with a 95% confidence level and a sample size of at least 225 based on the calculation made using the Sample Size Calculator software. The population of in-service contracted home-based care aides in Hualien County was reported to be 540 in 2019 [22]. Based on this information, the study reached out to all eligible home care service units in Hualien and distributed 412 copies of the questionnaire. Ultimately, the study collected 285 valid samples, resulting in a response rate of 69.2%. Data for the research was collected between December 2020 and January 2021. This study was reviewed and approved by the Research Ethics Committee of Buddhist Tzu Chi General Hospital (case number: IRB109-253-B).

The questionnaire comprises four sections, namely, intention to stay, personality traits, occupational burnout, and demographics and work experience. The scale of intention to stay was designed on the basis of the questionnaire of intention to stay developed by Yu et al. (1999) [23]. The scale consists of five items in total and the response options were rated on the 5-point Likert scale from 1 (always) to 5 (never), with the total score of the scale ranging from 5 to 25. A higher score indicates a higher intention to stay. The internal consistency of the scale reached 0.77. As for the personality trait scale, the Traditional Chinese version of the International English Big-Five Mini-Markers inventory translated by Deng et al. (2011) [24] was adopted, with a total of 40 items. The scale comprises five categories, namely, extroversion, openness, emotional stability, conscientiousness, and agreeableness. Each category consists of eight items, wherein the subjects were required to rate personality trait adjectives according to their personal situation. The scores of each item range from 1 (very untrue of me) to 9 (very true of me) and the total scores of each subscale range from 8 to 72. A higher score indicates a better alignment with the stated personality trait [24] and the internal consistency was measured as 0.91. In terms of the occupational burnout scale, this study adopts the Traditional Chinese version of the occupational burnout inventory developed by Yeh et al. (2008) with revisions incorporated on the basis of the Copenhagen Burnout Inventory (CBI) and the effort reward imbalance model (ERI) [25]. The scale comprises four subscales with 21 items in total: five on personal burnout, five on work-related burnout, six on client-related burnout, and five on over commitment to work. The response options were rated on the 5-point Likert scale from 0 (never) to 4 (always), with the total scores of each subscale ranging from 0 to 100. For the subscale on over commitment to work, a higher score indicates deeper involvement in work; for the subscales on personal burnout, work-related burnout, and client-related burnout, a higher score represents a higher level of burnout [25]. The internal consistency was measured as 0.90.

Demographics and work experience includes the following variables: age, gender (male / female), marital status (married / single), self-reported health status (good / worse), and health status 1 years ago (good / worse), family support level (supported / unsupported), seniority of home care aides (3 months–1 year / 1–2 years / 3–4 years / 5–6 years / 7 years or above), average salary a month (below 30,000 NTD / above 30,000 NTD), worked as home care aides under both the LTC 1.0 and LTC 2.0 policies (no - working as a home care aide for the first time / yes - prefer LTC 1.0 policy / yes - prefer LTC 2.0 policy), satisfaction of salary, promotion system, in-services education, reward and punishment, benefit system and current job (Yes / No).

In this study, SPSS Statistics 25.0 was utilized for data analysis to examine whether significant differences exist between the three constructs (namely, demographic variables and work experience, personality traits, and occupational burnout) and the intention to stay. Additionally, Person’s correlation was utilized to examine the relationships between variables. Variables with significant differences were subsequently subjected to hierarchical regression analysis, with the significance level herein set at 0.05.

## Results

A total of 285 home care aides were enrolled in this study. Results concerning the highest percentages for each demographic variable are detailed as follows: 38% of the subjects were between 41 and 50 years of age, 85.6% were females, the majority (83.8%) were married, the majority (96.5%) reported having obtained family support for their engagement in this profession, 34.4% have served as home care aides for 1–2 years. In terms of income, most home care aides (71.8%) earned more than 30,000 NTD per month, which was the income bracket with the most people. The study found that 65% of the participants were first-time home care aides. Of the remaining participants, 35% had worked as home care aides under both the LTC 1.0 and LTC 2.0 policies. 69.6% reported being compensated at a set hourly rate; 93.6% felt satisfied with their reward and punishment; and 77.5% felt satisfied with their current job as home care aides.

According to the study’s results, the highest score for occupational burnout among home care aides was obtained for burnout from over-commitment to work (mean: 43.49 ± 20.36), followed by personal burnout (mean: 35.79 ± 17.33), whereas client-related burnout received the lowest score (mean: 20.95 ± 15.43). Furthermore, the personality trait of agreeableness had the highest score (mean: 58.78 ± 10.64), whereas openness had the lowest score (mean: 44 ± 9.87). (Table 1)

**Table 1:** Sociodemographic characteristics of the home health aides (n = 285)

Demographics	n	%	Demographics	n	%
**Age** ^a^			**Family support for your job** ^a,^ ***		
21 ~ 30	22	7.7	Supported	275	96.5
31 ~ 40	64	22.5	**Seniority of home care aides** ^a^		
41 ~ 50	108	38.0	3 months − 1 years	76	26.7
51 ~ 60	69	24.3	1 – 2 years	98	34.4
≧ 61	21	7.4	3 – 4 years	30	10.5
Gender*			5 – 6 years	15	5.3
Female	244	85.6	> 7 years	66	23.2
Marriage**			Work status**		
Married	238	83.8	Part time	24	8.4
Health status***			Full time	261	91.6
Good	152	53.4	**Average salary/Month**		
Health status 1 years ago***			< NT 30,000	80	28.2
Good	167	58.6	> NT 30,000	204	71.8
**Education**			**Worked as a home care aide under both the LTC 1.0 and LTC 2.0 policies** ^a, *^		
Elementary school	17	6.0	No - working as a home care aide for the first time	182	65
Junior high school	46	16.3	Yes - prefer LTC 1.0 policy	9	3.2
Senior high school	149	52.7	Yes - prefer LTC 2.0 policy	89	31.8
College and above	71	25.1			
**Remuneration Payment**			**Satisfied of**		
By hourly rate	197	69.6	Salary***	167	58.6
By commission	64	22.6	Promotion**	173	62.5
By month	22	7.8	In-services education**	186	65.3
**Burnout**			Reward and punishment ^a,^**	265	93.6
Client-related burnout	20.95 ± 15.43		Benefits**	200	70.2
Over-commitment burnout	43.49 ± 20.36		Current job***	221	77.5
Work-related burnout	27.84 ± 15.79		**Personality traits**		
Personal burnout	35.79 ± 17.33		Extraversion	47.64 ± 10.27	
**Intention to stay**	21.44 ± 3.78		Openness	44 ± 9.87	
			Emotional stability	44.72 ± 7.65	
			Conscientiousness	54.23 ± 10.62	
			Agreeableness	58.78 ± 10.64	

Note: Statistics are displayed as Mean ± SD for the continuous variables and n (%) for the categorical variables

Use T-test or ANOVA analysis to examine the difference between variables and intention to stay, ^a^ were use non-parameter analysis of Mann-Whitney U Test or Kruskal-Wallis Test. * *p <* .05; ** *p <* .01; and *** *p <* .001.

The study investigated the correlation between occupational burnout, personality traits, and the intention to stay among home care aides. The results showed that all variables related to occupational burnout, including personal burnout, work-related burnout, and client-related burnout, were negatively correlated with the intention to stay, except for excessive commitment (*p* < .05). The study found that higher levels of personal, work, and client-related burnout were associated with lower intention to stay among home care aides; in terms of personality traits, all personality types except for openness (namely, extroversion, emotional stability, conscientiousness, and agreeableness) had significant positive correlations with the intention to stay (*p* < .05); higher levels of extraversion, emotional stability, conscientiousness, and agreeableness in personality were associated with a higher intention to stay among home care aides (Table 2).

**Table 2:** Correlation among intention to stay, burnout, and personality

	1	2	3	4	5	6	7	8	9	10
1. Intention to stay	1.00	− 0.33***	− 0.02	− 0.38***	− 0.26***	0.22***	0.10	0.19***	0.22***	0.30***
2. Client-related burnout		1.00	0.12	0.68***	0.44***	− 0.26***	− 0.20***	− 0.33***	− 0.35***	− 0.31***
3. Over-commitment burnout			1.00	0.16**	0.24***	0.09	0.12*	− 0.15**	0.12*	0.11
4. Work-related burnout				1.00	0.79***	− 0.27***	− 0.14**	− 0.35***	− 0.30***	− 0.24***
5. Personal burnout					1.00	− 0.21***	− 0.01	− 0.34***	− 0.21***	− 0.14**
6. Extraversion						1.00	0.45***	0.26***	0.43***	0.46***
7. Openness							1.00	0.24***	0.52***	0.39***
8. Emotional stability								1.00	0.48***	0.39***
9. Conscientiousness									1.00	0.63***
10. Agreeableness										1.00

Note: * *p <* .05; ** *p <* .01; and *** *p <* .001.

Based on Tables 1 and 2, variables achieving statistical significance were identified and sequentially entered into the hierarchical regression analysis model. The results are shown in Table 3.

Model 1 examined the relation between basic demographic information and intention to stay. The results revealed that being married, having family support, and better health status affected the intention to stay. The explanatory power of these factors in predicting intention to stay was found to be 12.9%. In Model 2, the study extended the analysis by including work experience. The results indicated that being female, being married, having better health status, being a full-time worker, and being more satisfied with promotion and current job affected better intention to stay (p < .05). The inclusion of these factors increased the explanatory power of the model by 10.5% compared to that in Model 1.The study conducted in Model 3 further expanded the analysis by adding burnout as a factor. The results demonstrated that being married, being a full-time worker, being more satisfied with promotion and current job, and having lower work burnout were all significant predictors of intention to stay (p < .05). The inclusion of these factors increased the explanatory power of the model by 4.5% compared to that in Model 2. Significant predictors for high intention to stay in model 4 were: married, full-time employment, satisfied with the promotion system and current job as home care aides on the whole, lower levels of work-related burnout, and agreeable personality type. In contrast, preference for the LTC 1.0 policy was found to be a significant predictor for low intention to stay (p < .05). The adjusted explanatory power increased by 1.5% compared with that in Model 3. On the variable level in model 4, among the influencing factors predicting the intention to stay, work-related burnout was found to have the greatest predictive power (β = −0.28), followed by agreeableness (β = 0.20) and satisfied with current job (β = 0.18) in that order. Work experience had the highest explanatory power among the factors analyzed in the study.

**Table 3:** Hierarchical regression analysis of related factors on intention to stay

Variables	Model 1		Model 2		Model 3		Model 4	
	Standardized *β*	*β*	Standardized *β*	*β*	Standardized *β*	*β*	Standardized *β*	*β*
**Demographics**								
Gender - female (ref: male)	1.22	0.11	0.12	1.28*	0.07	0.73	0.06	0.67
Marriage - yes (ref: no)	1.32	0.13*	0.17	1.69***	0.15	1.52**	0.15	1.56**
Family support - yes (ref: no)	1.62	0.19**	0.11	0.92	0.08	0.68	0.07	0.62
Health status in one year ago - better (ref: worse)	0.83	0.11	0.05	0.42	0.03	0.23	0.03	0.21
Health status - good (ref: worse)	1.29	0.17*	0.13	0.96*	0.08	0.61	0.08	0.60
**Work experience**								
Job - full time (ref: part time)			0.13	1.76**	0.12	1.66*	0.12	1.61*
Satisfied of salary (ref: no)			0.04	0.32	0.05	0.41	0.06	0.44
Satisfied of promotion (ref: no)			0.17	1.36***	0.14	1.11**	0.13	1.04*
Satisfied of in-services education (ref: no)			0.06	0.54	0.05	0.45	0.05	0.46
Satisfied of reward and punishment (ref: no)			0.03	0.27	0.04	0.31	0.02	0.18
Satisfied of benefits (ref: no)			-0.07	-0.60	-0.08	-0.69	-0.06	-0.47
Satisfied of current job (ref: no)			0.20	1.84***	0.19	1.72**	0.18	1.60**
Worked as a home care aide under both the LTC 1.0 and LTC 2.0 policies (ref: no)								
Yes - prefer LTC 1.0 policy			-0.12	-2.70*	-0.14	-3.03**	-0.12	-2.61*
Yes - prefer LTC 2.0 policy			-0.04	-0.31	-0.03	-0.26	-0.01	− 0.010
**Burnout**								
Work-related burnout					-0.29	-0.07**	-0.28	-0.07**
Personal burnout					0.16	0.03	0.15	0.03
Client-related burnout					-0.07	-0.02	-0.04	-0.01
**Personality**								
Extraversion							0.01	0.01
Emotional stability							-0.01	0.00
Conscientiousness							-0.06	-0.02
Agreeableness							0.20	0.07**
**R** ^**2**^	0.145		0.274		0.324		0.349	
**Adjusted R** ^**2**^	0.129		0.234		0.279		0.294	
**F**	9.02***		6.89***		7.13***		6.36***	

Note: ref. = reference group. * *p <* .05; ** *p <* .01; and *** *p <* .001.

## Discussion

Since the LTC 2.0 was officially put into effect, the concerned authorities are expected to improve the salary structure and professional image of care attendants, thereby enhancing their occupational identity and increasing the Taiwanese public’s intention to engage in home care services [2]. The statistics provided by the Taiwan government have indicated that although female have traditionally comprised the majority of home care aides, the percentage of males who are willing to work in this profession increased from 8.21% in 2016 to 14.91% in 2020 [26]. The results of this study on male home care aides were similar to the Taiwan government’s statistics on the percentage of male home care aides in 2020 [26].

During the decade when the LTC 1.0 was implemented, home care aides were principally compensated at a set hourly rate [27]. However, since the LTC 2.0 took effect, changes have been observed in remuneration payment. In addition to the conventional hourly pay method, which remains the most commonly adopted format, commission-based pay and monthly salary have also been offered, ranking as the second and third most frequently used formats respectively, according to the findings of this study. Moreover, a system of bonus payments for LTC personnel in remote rural areas was proposed in the LTC 2.0. Also, the government sent official letters to county governments across the country in 2018, announcing that the minimum monthly remuneration for home care aides should be raised to 32,000 NTD. In 2019, a professional polling firm surveyed remuneration payments offered by 385 home care service providers and found the average monthly remuneration for home care aides to be 38,498 NTD [28]. A comparative look at the payments received by monthly-salaried, full-time home care aides investigated in this study has revealed that the remuneration provided to home care aides in Hualien still has room for improvement [29].

In this study, work experience and condition were identified as the most influential factors for intention to stay, implying that these factors had the highest explanatory power among the variables examined in the study. Existing literature has suggested that the more adequate the working conditions, the lower the turnover intention [30]. This study has demonstrated that work patterns, satisfaction with job can be used to predict intention to stay among home care aides. In respect of work patterns, full-time home care aides displayed significantly higher intention to stay than their part-time counterparts [31]. The higher intention to stay in the former may result from their entitlement to more comprehensive benefits and better job security than those of the latter. In addition, the government of Taiwan officially added regulation in Long-Term Care Services Act in 2021, stipulating that the LTC providers, by contract, failing to enroll the LTC personnel employed by them into the labor insurance, national health insurance, and labor pension, among other types of insurance, shall be punished according to the same provisions and that if the providers still fail to comply with the requirement upon punishment, the providers may be suspended from being assigned. The inclusion of this article was intended to strengthen the right to job security and improve labor conditions for home care aides with an aim of increasing their employment intention. This may be the reason why full-time home care aides have a higher intention to stay than part-timers [21]. In addition, home care aides provided with a sound promotion system were shown to display a higher intention to stay. For Japan LTC system, the Japan National Council of Welfare Facilities for the Elderly has specified the career advancement path for home care aides, with a total of seven stages from first-time care aides to institution administrators, it is encouraging home care aides maintain their career path [32]. However, Taiwan has not yet established a unified standard for the promotion of home care aides. Currently, only a small number of private LTC providers have developed and implemented relevant systems independently. Despite this inadequacy, the government started organizing symposiums on the Care Attendant Empowerment and Training Quality Improvement Project – Home Care Aides Classification System at the end of 2021 [33]. This shows that the establishment of the promotion system for home care aides may contribute to increased intention to stay among home care aides.

This study has also found that higher job satisfaction led to higher intention to stay [34–36]. A study that employed analytic network process analysis was conducted to investigate the different factors affecting intention to stay, and it determined that satisfaction was the most crucial factor. Accordingly, this result suggests that if home care agencies develop targeted programs aimed at enhancing job satisfaction among home care aides, it could potentially increase their intention to stay in the profession, resulting in a more stable and high-quality workforce [36]. In addition, work burnout has been shown to have a statistically significant correlation with the intention to stay among home care aides, from which it can be inferred that work burnout has a crucial influence on intention to stay among home care aides. Therefore, home care agencies can potentially enhance retention rates by ensuring that they have an adequate number of home care workers and making timely adjustments to their workload to mitigate job burnout, which may lead to improved intention to stay [37, 38].

Moreover, previous studies have indicated that understanding employees’ personality traits can help companies delegate and arrange tasks more effectively [39]. A meta-analysis of 86 empirical studies has revealed that the personality traits of conscientiousness and agreeableness were the most consistently predictive factors in actual turnover decisions [40]. In another study, 536 nurses were surveyed through a questionnaire to investigate the relationship between personality traits and turnover, which found that respondents with higher levels of openness and emotional stability exhibited significantly lower intentions to stay [41]. A nursing study has indicated positive correlations between the four personality traits (excluding openness to experience) and intent to stay. Among the personality traits studied, agreeableness had the highest mean score [12]. The results of the study are consistent with past research that has found a positive relation between agreeableness and intention to stay. It also proposes that this relation may be due to the fact that individuals with high levels of agreeableness tend to be gentle, empathetic, cooperative, and willing to assist others, [42, 43]. which aligns well with the job nature of home care workers. Moreover, to be effective in providing home care services, workers need to possess qualities such as kindness, thoughtfulness, warmth, and high adaptability. Additionally, previous studies have confirmed a positive relation between agreeableness and engagement in service-oriented work [44]. Thus, assessing personality traits during the recruitment process can be beneficial in increasing the intention to stay of home care aides.

The research findings indicate that home care aides with work experiences, low work burnout and agreeable personality type would increase the intention to stay among this population. However, the research results may not be generalizable to the entire population of home care aides in Taiwan as the sample was recruited using purposive sampling method and only included home care aides from a remote rural area. In addition, the Hualien County is defined by the Ministry of Health and Welfare as an indigenous and remote area [45]. Previous survey studies have indicated that in Hualien County, at least 67.4% of home care workers are indigenous [46]. Therefore, the study sample may not be generalizable to the entire population of Taiwan. Moreover, the explanatory power of the model in this study was 29.4%, suggesting that some factors need to be clarified in future research. Despite this limitation, the study results are valuable as a reference for home care service providers when recruiting and designing related programs to improve intention to stay.

## Conclusion

This study set out to examine how the demographic and work experience, personality traits, and occupational burnout of home care aides are correlated with their intention to stay with the aim of understanding the factors influencing the intention to stay among this population. Furthermore, this study has concluded that increasing job satisfaction, establishing a sound promotion system, using personality traits as a criterion to select home care aides with a high level of agreeableness prior to recruitment, enhancing the professionalism and comprehensive of promotion system, and reducing work burnout are measures that may help home care service providers increase the intention to stay among home care aides.

## Data Availability

The datasets generated and analyzed during the current study are not publicly available because the data were collected through face-to-face interviews but are available from the corresponding author and participants on reasonable request.

## List of abbreviations

LTC: Long term care
LTC 2.0: Ten-year Long-term Care Plan 2.0
